# Dental Hints Department

**Published:** 1907-04

**Authors:** B. H. Teague

**Affiliations:** Aiken, S. C.


### ...DENTAL HINTS DEPARTMENT...

CONDUCTED BY B. H. TEAGUE, D. D. S., AIKEN, S. C., TO WHOM ALL CONTRIBUTIONS TO THIS DEPARTMENT SHOULD BE SENT

Heavy white unglazed paper, cut in 500 lots to fit brackets, is far cheaper than napkins.—Dr. G. A. Fletcher.

A few drops of glycerine added to iodine and aconite will prevent an annoying iodine blister.—Dr. G. A. Fletcher, The Frater.

A good soldering flux is made by mixing pulverized borax with vasaline.

White Japanese napkins fastened by two rubber bands to the head rest, so that the top one can slide off, keep head rest clean and antiseptic.—Dr. G. A. Fletcher, The Frater.

A finger bowl on bracket with one per cent. solution of carbolic acid, a few drops of budbear and a little toilet water makes an ideal hand sterilizer, sightly to patient and agreeable to the hands.—Dr. G. A. Fletcher, The Frater.

A simple and effective application for perspiring feet is to paint them at night with a mixture of equal parts of Monsell's solution and glycerine.—Clin. Med.

In mechanics the greatest stress is borne by a flat base at right angles to the direction of force. In approximal cavities the inlay should have a large, flat, cervical base. All other cavities should likewise present a flat seat at right angles to direction of greatest stress.—Items.

Before placing arsenic in a cavity that approaches the gum between the teeth, press the gum well down with a roll of cotton saturated with ammonia or dialized iron.

It is best to mix each color of porcelain on different vessels. The tile used for hearths are serviceable for the purpose. The tile should be numbered for the color. It will not be necessary then to cleanse it of the remains of the last mix.

GOLD SOLDER.—Take of any karat gold 23 parts, zinc 1 part and tin 1 part. Add the zinc first to the melted gold and immediately after the tin. Give the melted mass a shake or jar and pour quickly.

ALUMINUM IN PROSTHETIC DENTISTRY.—Dr. Booth Pearsall, of London, after several years of experience, recommends for persons subject to gout the use of aluminum base with ubber attachments.—The Dentists' Magazine.

It is well known that there is little difference between cohesive and non-cohesive gold, as it comes from the manufacurers, except that the non-cohesive has ammonia gas condensed on its surface.—DR. M. C. WARD, Register.

IODOFORM.—There are several substitutes for iodoform which act much in the same way and have not its objecttionable oder. The best of these is iodol which contains 90 per cent. iodin.—H. LEONARD DARRELL, Dental Record.

NIGHT OR DAY?—French therapeutists say medication by night is far more effective than by day, as absorption is more active and elimination slower.—Clin. Med.

Apomorphine is the best emetic if given hypodermatically; by the stomach it is not emetic, but emetine leaves nothing to desire, if given in grain doses in warm water.—Clin. Med.

THE POULTICE.—A hot poultice relieves pain but increases the growth of bacteria. An ice bag relieves pain and retards the growth of bacteria.—G. F. HEINEN, Clin. Med.

DENTISTS ORGANIZE.—The Jefferson County Dental society, of Birmingham, Ala., has been organized and the following officers elected: President, Dr. L. F. Luckie; vice-president,

Dr. George Reese; secretary and treasurer, Dr. J. A. Blue; corresponding secretary, Dr. L. Taylor; executive committee, Dr. Carmichael, Dr. Westbroow and Dr. Irwin. The object of the society is to promote the interest of the profession. Meetings will be held every two weeks when papers will be read and clinics given on all branches of dentistry. Quite a number of the leading dentists in the town have been behind the movement and it is expected to do great good.

To Clean Plates.—To remove calcareous deposits from rubber plates, drop them in a solution of muriatic acid and water; one part of acid to ten of water. Result is immediate.—Dr. G. G. Brown, Western Jour.

Putrescent Pulp.—On opening up a pulp-chamber in which there is a putrescent pulp giving out a most offensive odor, dip your broach in oil of turpentine and insert in canal; the odor will change almost instantly, most agreeably to both yourself and patient.—J. E. McDonald, Dominion Dental Journal.

Seriously Injured in Dental Office.—Miss Kittie Costello, a laundry girl in Denver, Colo., is in a dying condition caused by hypodermic injection of spirits of ammonia. Blood poisoning resulted and if the patient recovers, amputation of the arm will be necessary. It was done for the purpose of resuscitation from a faint.—Amer D. Jour.

The Odontagogon Law.—The skilful operator extracts more teeth with elevators than he does with forceps, for nearly every tooth that may be grasped with forceps may be saved. (Yet so-called ethical members of societies buy thousands of ounces of store anesthetics—and use them.)

The Apical Foramen.—The root of a permanent tooth is not fully formed, and the apical foramen smaller than the root-canal until the third or fourth year after the eruption of the tooth.—Dominion Dental Journal.

In fusing, porcelain tends to contract to a globular mass. Hence the cavity outline must not have a single angle but curves throughout.—Items.

Slow, long fusing at medium temperature to a final glaze, and slow cooling gives the toughest, most natural and homogeneous porcelain.—DR. C. EDSON ABERT, Items of Interest.

PREPARING SENSITIVE CAVITIES.—A comparatively painless method of cutting away a large body of sensitive dentin is to have the stones or burs run in water. I am able to do so-called heroic cutting with the stones run in water, so that the water is almost a running stream upon the bur or stone, and it can be run at a high rate of speed.—E. J. PERRY, Dental Review.

THE STERILIZATION OF DENTURES.—Sulphurous acid will absolutely deodorize and disinfect a denture and not merely cover the odor of a plate that has been worn in the mouth. Put a few drops in a little water and put the case in this at night. Cleanse with soap and brush in the morning.—I. KENNERLY RIDLEY, Journal British Dental Association.

VITIATED SALIVA.—Salivary excesses, because of gingivitis, produce digestive disorders either by the swallowing of vitiated saliva by pathologically diluted gastric juice, or cause indigestion, vomiting and, possibly, diarrhœa, by the loss of the saliva externally by expectoration. Digestion particularly is interfered with through insufficient insalivation.—W. T. MCLEAN, Medical Brief.

OBTUNDING SENSITIVE DENTIN.—Chlorid of sodium being used in the animal economy to promote endosmosis, why would it not, in solution with cocain, aid in conducting the latter through the dentin?—N. C. LEONARD, Dental Headlight.

FOR DANDRUFF.—One of the simplest washes for dandruff is the following: Chloral hydrate, 3 drs.; Glycerin, 5 drs; Water, 8 ozs. It will do more than any of the patent dandruff cures, and costs but a few cents.—Clin. Med.

MUMIFYING PASTE.—Para Formaldehyde; Thymol aa ½ dr.; Zinc oxide, 1½ dr.; Glycerine, q. s. to make a paste.—Exchange.

Antidote for Silver Nitrate.—The use of a solution of sodium chlorid, after the application of silver nitrate to any of the mucous membranes, will do away with the great discomfort to the patient resulting from the application of this drug.—Therapeutic Gazette.

To Clean Wax.—After melting and straining, boil the wax for 20 minutes in a solution composed of half-ounce oxalic acid to each quart of water. Old wax may be made cleaner than new. Two drops of oil of cassia added to each pound keeps wax aseptic and renders it less unpleasant to the patient.—Thos. Fletcher, Pacific Medical Journal.

For Rheumatism.—Try giving the juice of a lemon with a glass of hot water an hour before meals, and add a small teaspoonful of sulphate magnesia and we think you will get satisfactory results.—Clin. Med.

Rubber Plugger Handle.—A piece of rubber tubing the length of a gold plugger handle, and the proper size to snugly fit, will make a restful hand grip and prevent any sensation of writer's cramp. Try this on the nearly straight plugger, where you have to use considerable hand pressure.—Dr. W. L. Reed, Western Jour.

A Hot Brick.—A common brick is the best thing to warm rubber on before packing. It holds heat a long time and the rubber will not stick to it. Don't get a smooth one, just a common red brick, and you will wonder why such a good thing wasn't found out before.—O. F. Brigham, American Dental Journal.

The Matrix in Porcelain Inlay Work.—Gum camphor combined with paraffin seems to make the best material for packing the matrix before removing it from the cavity. Use the paraffin first, following it with pieces of gum camphor crowded in upon it. The paraffin is more adhesive, while the camphor gives stability. Both can be burned away without leaving any perceptible residuum.—E. Howard Babcock, Dental Cosmos.

Local Anesthetic.—Ethyl chloride as a freezing agent is a safe and reliable local anesthetic. Another is a mixture of equal parts of cocaine and brucine, one per cent. of each in water. Most of the difficulty with cocaine seems to be due to the use of too strong solutions.—Clin. Med.

Cementing Arsenic.—Mix the cement rather thin and place a small drop of it on a bit of paper and carry the paper to the cavity with the pliers; then press to place with a burnisher. The paper facilitates adjustment to place and prevents the cement adhering to the instrument.—C. B. Warner, Tri-State Quarterly.

Root Canal Filling.—After pulp removal under pressure anæsthesia I always leave a small quantity of mummifying paste (zinc oxid, alum and thymol) at the extreme end of the canal and then fill with gutta-percha points dipped in a saturated solution of thymol in oil of cinnamon. The cinnamon gradually evaporates, leaving a layer of thymol crystals, lining the root canal.—Wilfred E. Griffin, British Dental Journal.

Mixing Cement.—Remove the glaze of the glass cement slab, giving a slightly roughened surface. Use a hickory spatula; the fine fibre permits a millstone grind to the mixture of powder and liquid, insuring the breaking apart and turning over and around of all cement particles, giving a more even mixture and securing a more perfect chemical union.—D. R. Phillips, Northwestern Dental Journal.

If the glaze is sand papered from a porcelain crown, it is soon colored by the debris of the mouth and conforms more exactly to the color of the adjacent natural teeth. With the glaze on a slight difference is always readily detected.

Investing Crowns and Bridges.—Indifference and slovenly methods used in investing crowns and bridges produce too frequently unsatisfactory fitting bridges, and result in broken and checked facings. The habit of pushing a case into a soft moving mass of investment, which in turn rests on an uneven or soft surface, such as blotting paper, or even common paper, is bad practice. Proper size boxes, from the ordinary sandpaper disk boxes to well-selected ones of larger sizes, should be used. The boxes filled about two-thirds with investment material will firmly hold and securely incase every line and crevice of the invested bridge or crown. Save your sandpaper disk boxes; they are useful when investing a single crown or small bridges.—A. Percival Burkhart, Items of Interest.

Pain of Abscesses.—For the relief of the pain of a forming abscess or carbuncle, belladonna is more efficacious than opium. Ichthyol seems also to possess remarkable properties of similar effect. A most excellent prescription is: Ichthol 10.00, ext. belladonna 40.00, glycerin 50.00. Mix. Smear on flannel abundantly, apply to the sore, and cover with oiled silk, a pad of cotton and a bandage. It may be changed every three or four hours.—Clin. Med.

Strength of Porcelain.—Porcelain is about equal to enamel in edge strength. Hence joint between cavity margins and porcelain should be at right angles, if possible; direct bite on margins should be avoided; porcelain must have thickness for strength; use the blunt margin method in direct biting contact.

The blunt margin method, while successful in my hands, requires much testing, which we solicit for it. Where exposed to bite, slightly round exposed margins of tooth and inlay. This is done either in preparing cavity and packing body or with disks before setting. In distal cavities in cuspids and approximal cavities in bicuspids, slightly over-contour porcelain near occlusal margin rounding it off to the joint. If you do not do this, gentlemen, the force of mastication will break down these frail edges, leaving a rough crevice instead of a smooth rounded joint.—Dr. C. E. Abert, Items.

Replacing a Facing.—In repairing a facing on a bridge or crown the success of your operation depends greatly on the adaptation of the new facing to the old backing. This depends on getting your holes drilled in the right place and grinding the facing to fit. This can be very simply done by taking a pellet of cotton, saturate with alcohol, then rub it over a blue or black ink pad such as used for rubber stamps. Make a coating over the backing and press the facing to position. The ink will mark the place where it is necessary to grind. Wash thoroughly after facing is adapted and set with a slow setting cement. The pins may be secured by means of the Bryant system or by riveting.—C. J. HADLEY, Review.

Let us do away with the white sack coat of the barber and restaurant-waiter or make the similarity complete by wearing also the white apron. Substitute a Prince Albert coat made of brown linen or some dark washable material.

ECHAFOLTA.—Echafolta is anti-venom and if taken internally and applied at once to wounds caused by bites of venomous serpents, reptiles and insects, neutralizes the virus so that scarcely any swelling or inflammation follows the trauma. Every dental practitioner is daily using explorer, excavator and bur. Points, edges and blades are sharp and sometimes wound soft tissues near. Infection carried to such a trauma by careless operater or patient (often the latter) and a full rigged "blood poisoning" may sail in. Who but the dentist should "clear decks" for action? Echafolta, safe, sure. DR. A. C. HEWETT, Chicago, Ill., Dental Review.

METHOD OF SETTING AN INLAY. Instead of using direct pressure, as so many dentists do when they wedge an inlay to place, I put the inlay in position and draw a wide piece of tape over it, as I would in polishing a gold filling with a polisher. This keeps teetering the inlay from one end to the other, and if you keep going over it you will be surprised how much closer the inlay will become seated than if you use any amount of direct pressure. Direct pressure on the inlay will not squeeze out the cement, because the particles of cement rest on top of each other and cannot get away. I use a piece of tape twice the width of the inlay. W. H. TAGGART, Review.

PTYALISM. For tender, bleeding gums, from any cause, associated with fetid breath: Formaldehyde (40%) oz. i, thymol gr. x, tincture benzoini comp. oz. ij, alcoholis q. s. ad dr. iij. Teaspoonful in a glass of water as mouth wash, every three hours.—Medical News.

SILVER NITRATE WITH CEMENT FILLINGS.—A cement filling, zinc oxphosphate, placed upon a surface treated with silver nitrate, will, for some reason, last a great deal longer and will be a great deal better mass than the same mass not having the peculiar effect it gets from this film of silver albuminate. A great many, no doubt, have seen the effect of silver nitrate upon a surface which has been infected to a very slight depth. That in time will become a polished black surface and further decay will never result. Now, if in deeper cavities we can make partial preparation and apply silver nitrate and have it last longer than otherwise, it is worth knowing. DR. W. V. B. AMES, Review.

THE TEMPORARY PLATE. Where I can do so in the case of upper sets I prepare to extract the molars and second bicuspids some weeks before I insert the temporary set. The greatest shrinkage takes place during the first few weeks. I leave the front teeth for appearance and use, where they are of any use for mastication. Ladies who occupy public positions object to being left without any teeth whatever. When the gums where the molars and second bicuspids have been extracted are healed and the sharp points absorbed, I extract the remaining teeth and take the impression at the same sitting. Dental Digest.

THE RELIABILITY OF AMALGAM. During the past ten years, and a little over, I have kept up my work with this material, and during that time I can say that I know of many fillings that have been made by myself and others that have kept as clean margins, just as free from any discoloration of the dentin or enamel, as have any gold fillings and believe this may be made general. I do not claim thatthis can be done with the same regularity as can be done with gold; nor that it is as easy to do this in any ordinary method of working with amalgam—the manipulation of amalgam to produce regularly first-class results is more difficult than the manipulation of gold, therefore it is in that degree a less reliable material—but that the operations with amalgam which you perform for your patients can be improved, and vastly improved, I feel confident, without any possibility of doubt. G. V. Black. Dental Practice.

Gold Inlays. I use gold inlays almost every day, but I believe there is a place in operative dentistry for porcelain inlays, for gold inlays, and for gold fillings. I want to make clear just one point that I consider valuable for gold inlays. The gold inlay is especially valuable in such distal, or for that matter mesial, approximal, and occlusal cavities, in the posterior portion of the mouth where the teeth have lost their absolutely perpendicular position, and there is such a slant to the tooth that it makes the insertion of a gold filling a very difficult operation—I do not propose to say an impossible operation, for I have never seen a case that could not be properly filled with a gold filling, but it makes the insertion of a perfect gold filling so difficult that it is a dangerous expedient. Dr. M. L. Rhein, D. Mag.

Coloring Inlay. I first use the brown foundation: The idea is, that the more colors and layers that you get into the inlay, the less trouble you will have with discoloration on cementing, so that I first put a thin layer of brown in the floor of the matrix, not extending to the margin; on top of that I build out my contour, representing the dentine with the foundation body. Now, before this is baked, it probably corresponds in size and shape to the dentine of the tooth. On shrinkage, it is not as large as the dentine; then over this, on account of the yellow foundation not being a pleasing color to the eye, I place a yellow enamel. Now the yellow that is in the tooth comes from the dentine, and nothing but the dentine. All enamel is blue, and all normal dentine is yellow. Therefore, I select a yellow that corresponds to the dentine, and in doing this I match up the gingival margins and ascertain the shade of yellow to use. It is a brownish yellow, and not a pure yellow—a certain amount of brown mixed with it, and the colors used ordinarily are "C" or "D" on the Brewster shade guide.—A. W. STARBUCK, Wes. Journal.

WEIGHT EQVIVALENTS

To convert grains into grammes multiply by.......... 0.065
To convert grammes into grains multiply by.......... 15.5
To convert drachms into grammes multiply by........ 3.9
To convert ounces (avoir.) into grammes multiply by 28.4
To convert pounds (avoir.) into grammes multiply by..............................................453.6

MEASURE EQUIVALENTS.

To convert cubic centimeters into grains multiply by 15.5
To convert cubic centimeters into drachms multiyly by.................................................. 0.26
To convert cubic centimeters into ounces (avoir.) multiply by.............................................. 9.036
To convert pints into cubic centimeters multiply by 473.
To convert liters into ounces (avoir.) multiply by 35.3
To conver gallons into liters multiply by ............ 3.8

—Scientific American.

FOR SENSITIVE CAVITIES.—There was a discussion at the August meeting of the American Dental Society of Europe on this subject, in which Dr. Bogue, of New York, told us he used pure carbolic acid first in the cavity and afterward chloride of zinc. I have been experimenting for some months with peroxide of sodium, and now having used Dr. Bogue's hint about using carbolic acid first, I find I can control very troublesome cases with not too much previous pain. For three or four years I have used very finely powdered peroxide of sodium for relieving sensitive places on the necks of teeth, or spots rendered sensative by contact with gold bands supporting plates. This led me to use it occasionally for troublesome cavities. My present method is, flood the cavity with pure carbolic acid aud wait a minute or so, then put into the cavity a little finely powdered peroxide of sodium without wiping out the acid. This causes some pain. Add a few more grains and commence to carefully excavate. If the rubber dam is not used, care must be taken of the gum.—Dr. Henry I. Moore, Review.

Abscessed Teeth.—After the canals have been permanently filled, and if a painless operation is essential, it is my custom to inject into the gum a saturated solution of cocaine. First, giving the patient 10 gtt. of volasem, which is a perfect antidote, and without which no more than a 2 per cent. solution of cocaine should be used. Even a solution of this strength should be guarded by a heart and respiratory stimulant.

After an elapse of a minute or two, plunge a drill, such as the one I have shown, in direct line with the apex of the root. If there is more than an acute inflammation, a cavity will be found in the process at the apex of the root. The amount of operating to be done will depend upon the extent of the cavity and the condition of the root and surrounding tissues.

If the inflammation is slight, the mere giving vent to the congestion by the blood-letting which follows the withdrawal of the burrs is generally sufficient to effect a cure. The burr, however, should always be run over the end of the root to break up the peridental membrane and allow any pus it may contain to escape.

This simple operation is usually all the treatment required. The size of thd opening necessary to be made through the gum and process depends upon the amount of disease present, and whether or not a gauze dressing is necessary.—Dr. Curtis, Dental Surgeon.

An Effective Ligature.—In cases where the clamp is objectionable for the retention of the rubber dam, and where the ordinary floss is not sufficiently bulky to prevent the rubber from drawing over it, a most admirable method of using the ligature is to first pass the floss through two pieces of rubber tubing, allowing one piece on the buccal and one on the lingual side of the tooth. This is much preferable to stringing beads on the ligature or using other agents, as already suggested. The tubing should be the smallest size sold at the rubber stores, the kind used for slipping over the bows of glasses where they rest on the ears. To insure against leakage, drop a little sandarac varnish between the tubing and the enamel on buccal and lingual sides. Dr. E. M. S. Fernandez, Review.

Injuries from Injections. I have seen so much injury to the gums, alvolar process and bone, resulting from hypodermatic injections, that I would prohibit the use of cocaine in my office if I thought the trouble arose from the use of cocaine per se. But I am of the opinion that the trouble frequently comes from septic matter carried into the tissues beyond by the needle rather than by the irritating properties of the drug itself. A thorough asceptic needle may, and often does, carry infectious material from the mouth into the the tissues, and thus cause serious trouble. It is a well known fact that infectious material in the mouth, and about the necks of the teeth, is often carried into the general system by the beaks of the forceps in extraction, causing serious trouble. It is also well known that cocaine is a very unreliable drug in its effects upon different individuals. But its injudicious, indiscriminate and improper use has doubtless caused a very large part of the trouble that has been blamed upon the drug itself. Dr. C. P. Pryun, Review,

Non-Corrosive Sterilizer for Dental Instruments.—Requirements: In form, liquid; in action, rapid, certain, penetrating, bland, volatile, cleanly, uncontaminating, non-toxic, non-irritant, antiseptic, germicidal, portable, inexpensive, analgesic, easily prepared, convenient to apply to each instrument, each time used, and non-corroding.

R Specific echfolta (Lloyds)............1½ oz.
Alcohol deodorized..................½ oz.
Glycerin Pur ......................Gtts. x
Ol. Rose Geranium...............Gtt. ij
Mur. cocaine (coarse crystals) Grs. xxxviij
Acid carbolic (crystals).............grs. xx
M. Sig. as directed.

Procure a glass bon-bon dish three inches in diameter by one and one-half inches in height; sides oval; in use pour off compound from well corked bottle enough to cover a bur head, drill, excavator or searcher point, and before or after using either dip in the compound, each time either is used, and at night before laying away. Forceps points wet with the lotion will become instantly sterilized. Two or three drams of the compound will last a month if evaporization is met by the addition of alcohol and the dish is kept covered when not in use. A piece of thick rubber-dam makes a good cover. The dish and two ounces of the compound need not cost more than a dollar. I have used the preparation for years and know its efficiency. Its value is beyond price.—Dr. A. C. Hewett, Review.

